# Supplementary material for: The Drosophila melanogaster Y-linked gene, WDY, is required for sperm to swim in the female reproductive tract
Source: Commun Biol. 2024 Jan 12;7:90. doi: 10.1038/s42003-023-05717-x (PMC10786823; doi:10.1038/s42003-023-05717-x)
Supplement: Supplementary file 2 — Supplementary Information [file 42003_2023_5717_MOESM2_ESM.pdf]

SUPPLEMENTARY MATERIALS

Supplementary Table 1: Drosophila Stocks

**Full Genotype**	**Source**	**Notes**
y[1] w[*] P{y[+t7.7]=nos-phiC31\int.NLS}X; PBac{y[+]-attP-9A}VK00027	BL #35569	phiC31/attP9A line used to make guide-expressing flies, from Rainbow Transgenics
y[1] w[*]; wg[Sp-1]/CyO; Dr[1]/TM3, Sb[1]	BL #59967	for balancing transformants
w[*]; TM3,Ser /TM6B, Tb	Wolfner Lab Stocks	for balancing transformants
w[1118]/TI{Disc\RFP[tdTom.3xP3]=attP}AByG	BL #78567	labelled Y chromosome
C(1;Y)3, In(1)FM7, v[Of]/C(1)M4, y[1]/0	BL #988	Source of compound X^X
C(1;Y)1, y[1]/0; ry[506]	BL #4408	Source of compound X^Y
w[*]; {U6:3-gRNA 3xWDY, w+}attP-9A / TM3	this study	transformant, expresses *WDY* and *ebony* guides ubiquitously
w Protamine-GFP, w+ ;; Bam-Gal4, UAS-Dicer	Chen and McKearin 2003, Manier et al 2010	Combined into one stock by Caroline Sartain
y[1] w[118] ; {nos-Cas9}attP2 / TM3	Kondo et al 2020	nos-Cas9 on III
yw ; {UAS-WDY RNAi}attP	VDRC_109045	WDY RNAi Line
yw ; {UAS-kl3 RNAi}attP	VDRC_109151	Kl-3 RNAi line
yw ; attP VIE-260B	VDRC_60100	RNAi Control Vie260
C(1;Y)1, y[1]/C(1)M4, y[1]/TI{Disc\RFP[tdTom.3xP3]=attP}AByG, WDY[F8]	this study	WDY F8 allele
C(1;Y)1, y[1]/C(1)M4, y[1]/TI{Disc\RFP[tdTom.3xP3]=attP}AByG,	this study	WDY C104 allele
C(1;Y)1, y[1]/C(1)M4, y[1]/TI{Disc\RFP[tdTom.3xP3]=attP}AByG, WDY[C3]	this study	WDY C3 allele
C(1;Y)1, y[1]/C(1)M4, y[1]/TI{Disc\RFP[tdTom.3xP3]=attP}AByG, [C7]	this study	Control C7 allele
C(1;Y)1, y[1]/C(1)M4, y[1]/TI{Disc\RFP[tdTom.3xP3]=attP}AByG, [G107]	this study	Control G107 allele

Supplementary Table 2: Guide Sequences

Guide Name	Guide Sequence	PAM	Length	Reference
ebony	GCCACAATTGTCGATCGTCA	AGG	20	Kane et al. 2017
WDY 2.4n	AAGTATAGAGGTCGCTGCCC	TGG	20	
WDY 2.5n	ATAGTTTGGGATCCTTGGAC	AGG	20	
WDY 2.6n	CATAACATTGTGGTAACAGG	AGG	20	

Supplementary Table 3: Primer Sequences

#	Name	Length	Sequence	Comments
**CRISPR plasmid construction primers**				
YH114	e_CP_1F	59	TTCCCGGCCGATGCAGCCACAATTGTCGATCGTCAGTTTAAGAGCTATGCTG	[pAC-U63-tgRNA-Rev tRNA Overhang] + [*ebony* guide] + [pMGC core primer]
YH115	WDY_CP_1R	37	CCTGTTACCACAATGTTATGTGCACCAGCCGGGAATC	[pMGC tRNA primer] + [WDY2.6n guide]
YH116	WDY_CP_2F	44	CATAACATTGTGGTAACAGGGTTTAAGAGCTATGCTGGAAACAG	[WDY2.6n guide] + [pMGC core primer]
YH117	WDY_CP_2R	37	GGGCAGCGACCTCTATACTTTGCACCAGCCGGGAATC	[pMGC tRNA primer] + [WDY2.4n guide]
YH118	WDY_CP_3F	44	AAGTATAGAGGTCGCTGCCCGTTTAAGAGCTATGCTGGAAACAG	[WDY2.4n guide] + [pMGC core primer]
YH119	WDY_CP_3R	57	TTCCAGCATAGCTCTTAAACGTCCAAGGATCCCAAACTATTGCACCAGCCGGG	[pMGC tRNA primer] + [WDY2.5n guide] + [pAC-U63-tgRNA-Rev Core
***WDY* genomic target site primers**				
ST20	WDY_3F	27	TGAGATGGTATCTTGCGTTTACTTTTC	Tm = 60°
ST21	WDY_3R	27	ACTTCTTGGTCTGGCATTATACTCATA	Tm = 60°
YH96	tgRNA_3F	20	GCCTCGAGTTAACGTTACGT	Tm = 58°, used to check plasmid and transgenic lines
YH97	tgRNA_2R	21	CGTCAACGGAAAACCATTGTC	Tm = 58.4°, used to check plasmid and transgenic lines

Supplementary Table 4: Alleles Generated

Allele	Characteristics of the allele
Y_Tomato	unedited marked Y chromosome
Control_G107	went through crossing scheme but showed no changes at target site
Control_C7	went through crossing scheme but showed no changes at target site
WDY[F8]	547bp deletion between guides 2.4n-2.5n
WDY[C104]	545bp deletion between guides 2.4n-2.5n
WDY[C3]	443bp deletion between guides 2.6n-2.5n + 3bp change at guide 2.4n

Supplementary Table 5: Sterility Test of *WDY*

Genotype	Allele	Fertile	Sterile
Control	tdTomato	18	0
Control	C7	17	0
Control	G107	17	0
WDY	F8	0	19
WDY	C104	0	19
WDY	C3	0	19
Control	RNAi	18	1
WDY	RNAi	5	15
kl-3	RNAi	0	10

Supplementary Table 6: *D. melanogaster* WDY Annotations

Domain	Motifs	Sequence
EF Hand	EF Hand Odd	YSSFLNTPERHVGIDELR
	EF Hand Even	MYTRLFLKINQNRDFKVDWNEFVSYLIFGF
WD-40	WD40-6 partial	MVRKS
	WD40-1	ICCLALLKAKSDQVPIDEVTETINFSFGGEDSPEASGMWVTASHEGMMRFWTSHMEPIRTASS
	WD40-2	SYAFFNNGKVHSKLILGDYAGNVRVLSYSPHLRGPFQAKPGAALIEVVWSDILRGKIPQFFPKEY
	WD40-3	SCVYFSLHMNALFASAEYRNTKKYRGRCPGMIMVTYDERSNF
	WD40-4	VSTFFVAESHNIVVTGGPDTFVRIWDVYIPTEPSAIL
	WD40-5	GHNGGIVLVFVQPEENKVYSVDYQKIIKVWDLHEHTLLQTY
	WD40-6 partial	TYYYHSHLRELVVAGRKLISIK
WD-40	WD40-7	THAAPVSVVLYNRLFRNIVSCGLDSYIIVWDPWTGRRKIIM
	WD40-8	EIIDIEITAACFDPLEQFLLTGARDGSLKIWNYNNAVVVRNMS
	WD40-9 partial	QEVTAVIWVVDRILAMGWDRQVTEFNDVVGR
	WD40-10	FHTDDITCADVKLGEGVVTATYSGEIIFWKLETGQPYRRYSV
	WD40-11	QAVLFLQTRPQTLKHGSVFISLDTGFIQVYSHHQRGGYIVEFL
	WD40-12	KTGDCVLTMCTDRKNRYLYTGTAFGYIKVWYIVNFCVPESEKTHVCMPKLRLEFIFLRKELFMTRAKRAVRNQAEPLLVSSYKG
	WD40-13	HLKAINSIAFINLPKIIFTGSHDYSCRLWTQGGRYLGT

Supplementary Table 7: Source of sequences of WDY orthologues

		Group				
**Species**	**Source of DNA Sequence**	***D.melanogaster***	***D.melanogaster*, no *D.triauraria***	***D.obscura***	***D.virilis***	**D.montium**
D.melanogaster	NM_001316659.1 (full length)	●	●			
D.sechelia	Chang et al, 2022, eLife 11:e75795 (full length)	●	●			
D.simulans	Chang et al, 2022, eLife 11:e75795 (full length)	●	●			
D.mauritiana	Chang et al, 2022, eLife 11:e75795 (full length)	●	●			
D.erecta	Genbank: HQ852741.1 (missing N-terminus)	●	●			
D.yakuba	Genbank: BK006450.1 (missing N-terminus)	●	●			
D.asahinai	Extracted from VNJZ01001055.1 via Exonerate (EF-hand only)					●
D.lacteicornis	Extracted from VNKF01012331.1 via Exonerate (EF-hand only)					●
D.tani	Extracted from VNJO01004106.1 via Exonerate (EF-hand only)					●
D.auraria	Extracted from VNJW01007069.1 via Exonerate (EF-hand only)					●
D.triauraria	Extracted from CM024333.1 via Exonerate (full length)	●				●
D.pectinifera	Extracted from VNKC01003049.1 via Exonerate (EF-hand only)					●
D.anomelani	Extracted from JAEIIZ010011789.1 via Exonerate (EF-hand only)					●
D.ananassae	Genbank: EU362855.1 (full length)	●	●			
D.miranda	Extracted from NC_030306.1 via Exonerate (full length)			●		
D.persimilis	Extracted QMET02000011.1 from via Exonerate (full length)			●		
D.pseudoobscura	Genbank: BK006447.1 (full length)			●		
D.obscura	Extracted from JAECWW010000199.1 via Exonerate (full length)			●		
D.guanche	Extracted from OUUW01000006.1 via Exonerate (full length)			●		
D.subobscura	Extracted from CM017786.1 via Exonerate (full length)			●		
D.willistoni	Genbank: BK006446.1 (full length)			●		
D.virilis	Genbank: BK006445.1 (full length)				●	
D.novamexicana	Extracted from QMEP02000025 via Exonerate (full length)				●	
D.arizonae	Extracted from LSRM01000002.1 via Exonerate (full length)				●	
D.mojavensis	Genbank: BK006444.1 (full length)				●	
D.navojoa	Extracted from LSRL02000078.1 via Exonerate (full length)				●	
D.hydei	Extracted from QMEQ02000040.1 via Exonerate (full length)				●	
D.grimshawi	Genbank: BK006443.1 (full length)				●	

Supplementary Table 8: Tests of Positive Selection on Full-Length WDY

We used PAML's codeml program to analyze nucleotide coding DNA sequences for signatures of positive selection. Model comparisons were performed between null models without positive selection and alternative models that include positive selection (M1a v M2a, M7 v M8, and M8a v M8). Model comparisons where the null is rejected are highlighted in gray. Although for the melanogaster clade, the null hypothes was rejected for the M7 v M8 comparison, the null hypothesis was not rejected for the M8a v M8 comparison. The observed discrepancy in rejection of the null between both model comparisons likely indicates a false positive detection of selection by M7 v M8 comparison due to a subset of sites in WDY undergoing neutral evolution in the melanogaster clade. M7 restrics all categories of sites to have an omega <1, while M8a allows a subset of sites to have an omega = 1. Since M8a accounts for neutral evolution, it can be a more stringent null model for determining signatures of positive selection. Codeml analysis using all available WDY sequence does not show evidence of WDY undergoing positive selection. When analyzing all WDY sequences for positive selection, no selection was detected.

Group	Model Comparison	Null lnL	Alternative lnL	2x(ΔlnL)	df	p-value	omega	p(sites)	BEB sites with P>0.9
D. melanogaster	M1a v M2a	-10017.35696	-10017.35696	0	2	1			
	M7 v M8	-9989.681483	-9986.668286	6.03	2	0.049	1.18	0.01	
	M8a v M8	-9986.711538	-9986.668286	0.09	1	0.76			
D. melanogaster, no D. triauraria	M1a v M2a	-8507.453522	-8507.453522	0	2	1			
	M7 v M8	-8498.558319	-8496.304226	4.51	2	0.104			
	M8a v M8	-8496.664448	-8496.304226	0.72	1	0.4			
D. obscura	M1a v M2a	-9579.376445	-9579.376445	0	2	1			
	M7 v M8	-9563.499663	-9562.97558	1.05	2	0.59			
	M8a v M8	-9562.97558	-9562.97558	0	1	1			
D. virilis	M1a v M2a	-12026.53699	-12026.53699	0	2	1			
	M7 v M8	-12017.25419	-12015.6323	3.24	2	0.2			
	M8a v M8	-12015.67787	-12015.6323	0.09	1	0.76			
**All Sequences**	M1a v M2a	-24300.91375	-24300.91377	0	2	1			
	M7 v M8	-23949.18667	-23949.19039	0.01	2	1			
	M8a v M8	-23949.19039	-23949.19039	0	1	1			

Supplementary Table 9: Tests of Positive Selection on EF Hand Domain of WDY

We restricted selection analysis to the EF hand domain and incorporated additional sequences from species in the montium subgroup for which full-length sequences were not available. We, again, used PAML's codeml program to analyze nucleotide coding DNA sequences for signatures of positive selection. Model comparisons were performed between null models without positive selection and alternative models that include positive selection (M1a v M2a, M7 v M8, and M8a v M8). Model comparisons where the null hypothesis was rejected are highlighted in gray. For the M7 v M8 comparison, the null hypothesis was rejected only for the D.melanogaster group when *D. triauraria* was included. However, the null hypothesis was rejected for the M8a v M8 comparison, indicating a likely false positive caused by neutral evolution.

Group	Model Comparison	Null lnL	Alternative lnL	2x(ΔlnL)	df	p-value	omega	p(sites)	BEB sites with P>0.9
D. melanogaster	M1a v M2a	-551.231303	-551.231306	0	2	1			
	M7 v M8	-557.146028	-551.401094	11.5	2	0.0032	1.7	0.015	
	M8a v M8	-551.654089	-551.401094	0.51	1	0.48			
D. melanogaster, no D. triauraria	M1a v M2a	-458.980644	-458.980645	0	2	1			
	M7 v M8	-459.812004	-459.107967	1.41	2	0.49			
	M8a v M8	-459.107993	-459.107967	0	1	1			
D. montium	M1a v M2a	-463.42136	-463.42136	0	2	1			
	M7 v M8	-463.072468	-463.072611	0	2	1			
	M8a v M8	-463.072611	-463.072611	0	1	1			
D. obscura	M1a v M2a	-740.774614	-740.774614	0	2	1			
	M7 v M8	-740.552849	-739.603289	1.9	2	0.37			
	M8a v M8	-740.13037	-739.603289	1.05	1	0.3			
D. virilis	M1a v M2a	-686.713995	-686.713995	0	2	1			
	M7 v M8	-686.079771	-686.080456	0	2	1			
	M8a v M8	-686.079866	-686.080456	0	1	1			
**All Sequences**	M1a v M2a	-1856.81276	-1856.81276	0	2	1			
	M7 v M8	-1809.417365	-1809.418055	0	2	1			
	M8a v M8	-1809.418031	-1809.418055	0	1	1			

Supplementary Table 10: Branch-site Test of Positive Selection on Full-Length WDY

We used PAML's codeml program to run a branch-site test for positive selection. Model comparisons were performed between null model (M0) without positive selection and alternative model (M2) that include positive selection on the branch where WDY moved to the Y-chromosome. Although the model passes, positive selection was not detected on the branch leading to species where WDY is Y-linked.

**Results of Branch Site Test on All Sequences**	
**Model Comparison**	M0 v M2
**Null Model**	-24534.935013
**Alternative Model**	-24521.014917
**Chi-Square**	27.84
**df**	1
**p-value**	<.00001
**dN/dS - Y-linked branch**	0.0136
**dN/dS - autosomal branch**	0.056

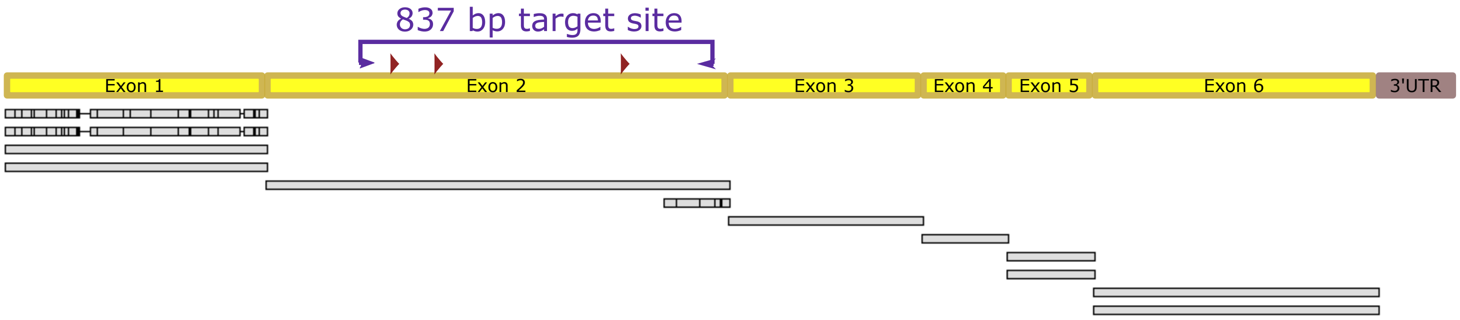

Supplementary Figure 1: CRISPR target site on exon 2 of *WDY* had no identifiable duplicates. Exon structure of *WDY* (introns not shown) with CRISPR target site marked. Purple arrows indicate primers, red triangles indicate guides. Additional copies of *WDY* regions on the Y chromosome are displayed as grey bars underneath the *WDY* schematic.

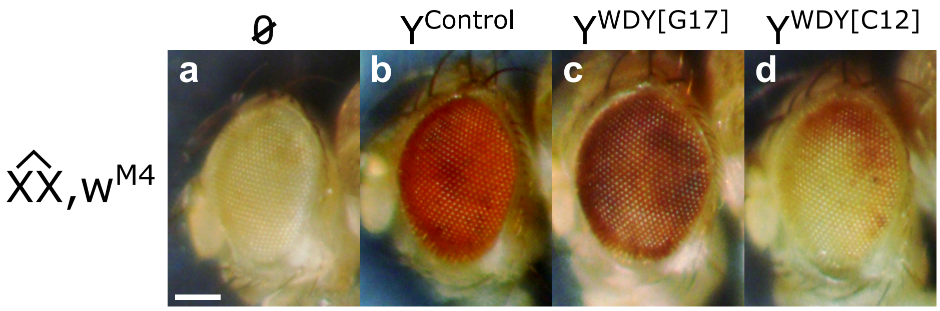

Supplementary Figure 2: Position effect variegation observed in some lines after CRISPR editing at *WDY*. Adult eyes of females with compound-X containing the position effect variegation marker, w^{M4}, and no Y chromosome (a), an unedited Y chromosome (b), or CRISPR-edited Y chromosomes G17 (c) or C12 (d). Scale is 100 µm.

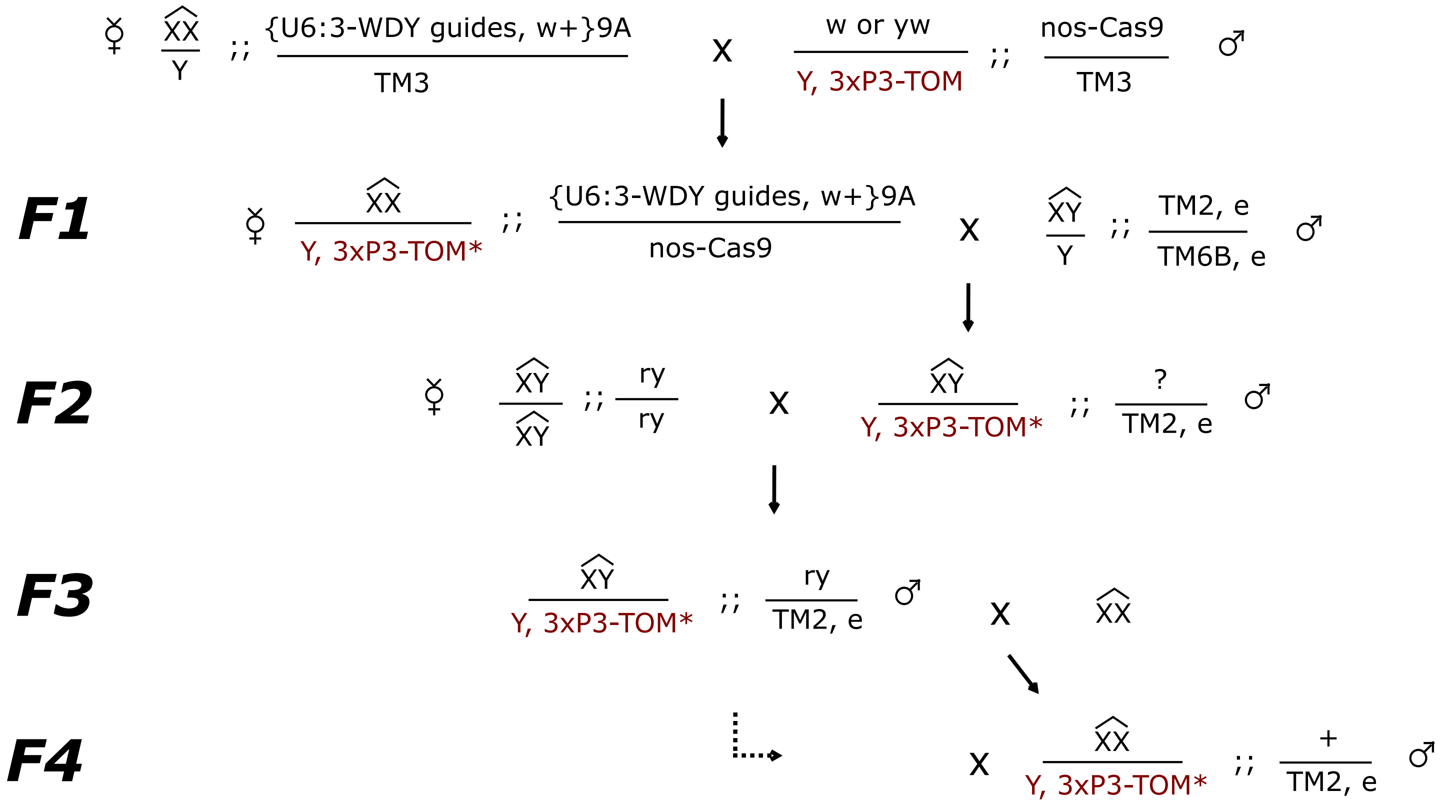

Supplementary Figure 3: Crossing scheme for inducing CRISPR, then balancing the edited Y chromosome using compound-X and attached X-Y chromosomes.

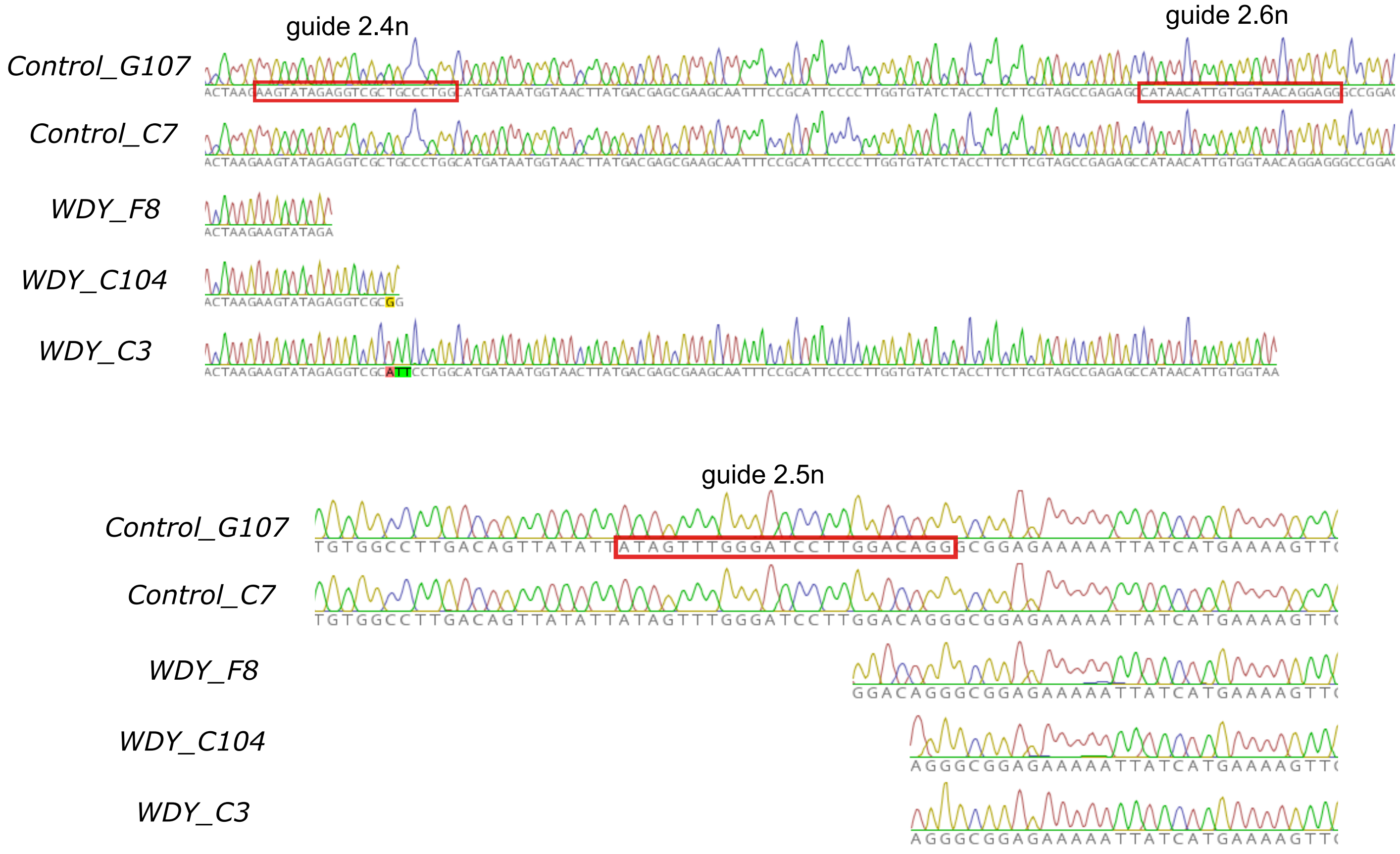

Supplementary Figure 4: Sequencing results in target region for *WDY* and control alleles used in this study. Guide sites are marked with red boxes. Nucleotide changes are highlighted. Gaps represent deletions.

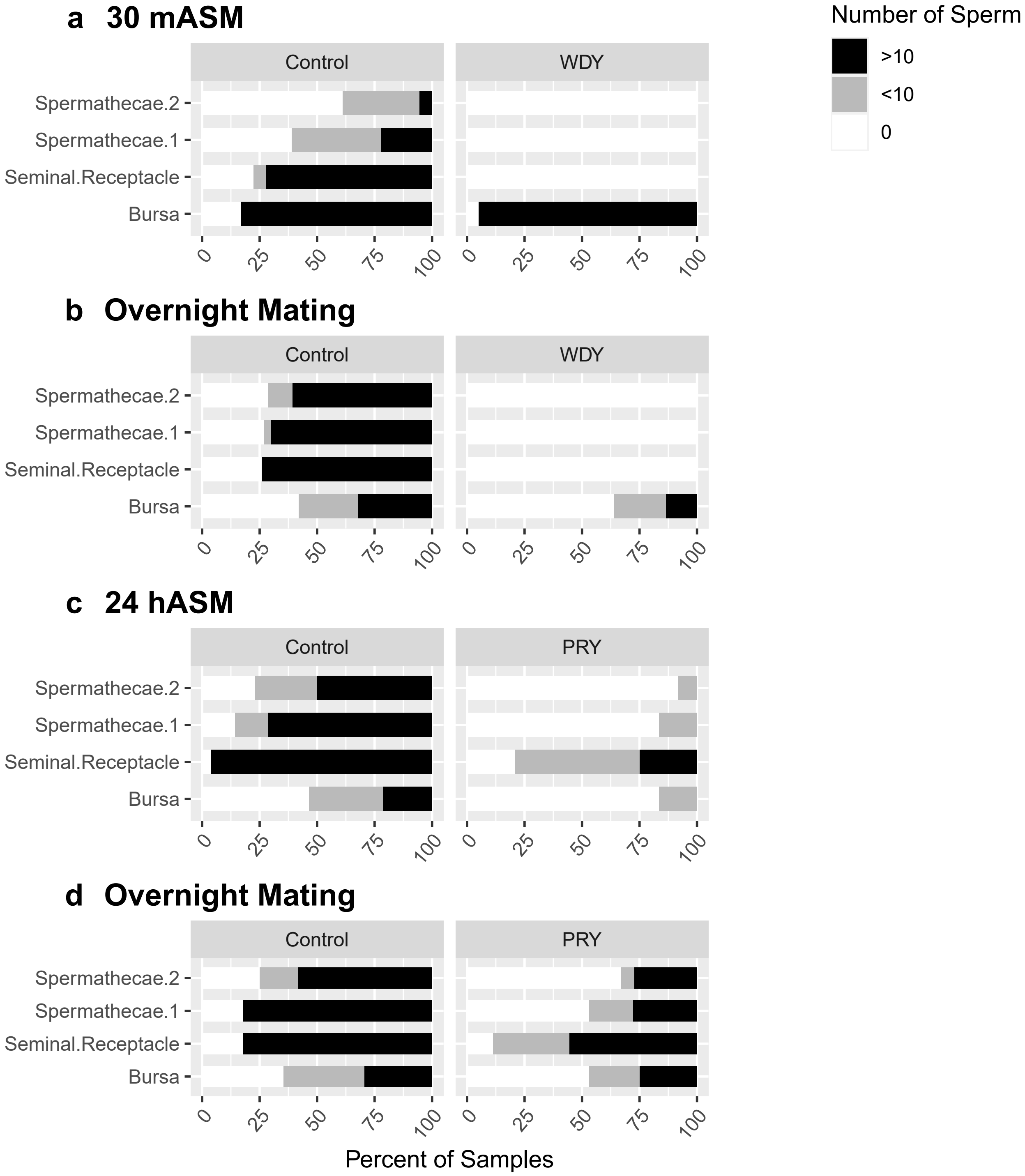

Supplementary Figure 5: Distribution of *WDY* and *PRY* mutant sperm in the female reproductive tract after mating. (a) Quantification of *WDY* mutant sperm's distribution in the female reproductive tract 30 mASM (p=3.6e-8, Asymptotic Linear-by-Linear Association Test for difference in sperm distribution). (b) Quantification of *WDY* mutant sperm's distribution in the female reproductive tract after overnight mating (p2.2e-16, Asymptotic Linear-by-Linear Association Test for difference in sperm distribution). (c) Quantification of *PRY* mutant sperm's distribution in the female reproductive tract 24 hASM ($p<0.001$, Asymptotic Linear-by-Linear Association Test for difference in sperm distribution). (d) Quantification of *PRY* mutant sperm's distribution in the female reproductive tract after overnight mating ($p<0.001$, Asymptotic Linear-by-Linear Association Test for difference in sperm distribution).

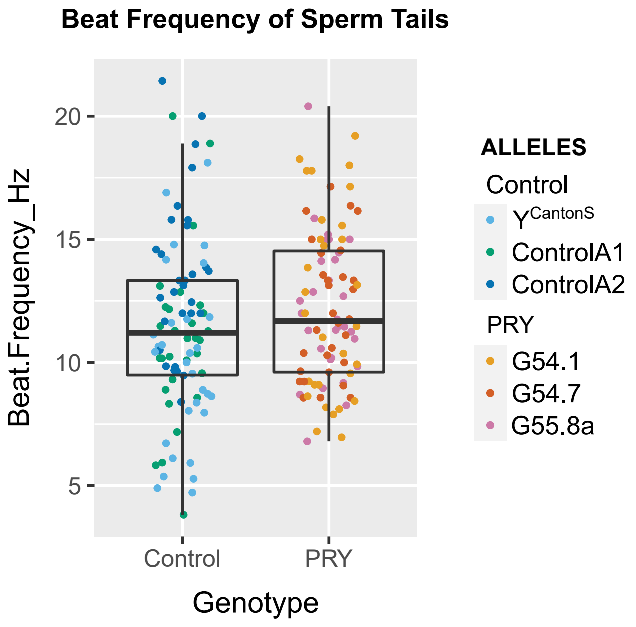

Supplementary Figure 6: Beat frequency of *PRY* mutant sperm in the seminal vesicle.
Quantification of tail beat frequency of *PRY* and control sperm dissected from seminal vesicles (n= 30 sperm tails per Allele, p-value =0.5316, Likelihood Ratio Test). Boxplots show median, first and third quartiles, and values within 1.5X the interquartile range for each genotype.
