# Supplementary material for: The Drosophila melanogaster Y-linked gene, WDY, is required for sperm to swim in the female reproductive tract
Source: Commun Biol. 2024 Jan 12;7:90. doi: 10.1038/s42003-023-05717-x (PMC10786823; doi:10.1038/s42003-023-05717-x)
Supplement: Supplementary file 3 — Description of Additional Supplementary Files [file 42003_2023_5717_MOESM3_ESM.pdf]

Description of Additional Supplementary Files

File name: Supplementary Movie 1
Description: Sperm dissected from seminal vesicles of control males.

File name: Supplementary Movie 2
Description: Sperm dissected from seminal vesicles of WDY males.

File name: Supplementary Movie 3
Description: Sperm dissected from the uterus of females mated to control males.

File name: Supplementary Movie 4
Description: Sperm dissected from the uterus of females mated to WDY males.

File name: Supplementary Movie 5
Description: Control sperm heads in the posterior testes.

File name: Supplementary Movie 6
Description: WDY sperm heads in the posterior testes.

File name: Supplementary Movie 7
Description: Control sperm heads in the seminal vesicle.

File name: Supplementary Movie 8
Description: WDY sperm heads in the seminal vesicle.

File name: Supplementary Movie 9
Description: Control sperm heads in the ejaculatory duct.

File name: Supplementary Movie 10
Description: WDY sperm heads in the ejaculatory duct.

File name: Supplementary Movie 11
Description: Control sperm heads in the uterus.

File name: Supplementary Movie 12
Description: WDY sperm heads in the uterus.

File name: Supplementary Movie 13
Description: PRY sperm heads in the uterus.
